# Increasing Adiposity Is Associated With QTc Interval Prolongation and Increased Ventricular Arrhythmic Risk in the Context of Metabolic Dysfunction: Results From the UK Biobank

**DOI:** 10.3389/fcvm.2022.939156

**Published:** 2022-06-29

**Authors:** Kiran Haresh Kumar Patel, Xinyang Li, Xiao Xu, Lin Sun, Maddalena Ardissino, Prakash P. Punjabi, Sanjay Purkayastha, Nicholas S. Peters, James S. Ware, Fu Siong Ng

**Affiliations:** ^1^National Heart and Lung Institute, Imperial College London, London, United Kingdom; ^2^Imperial College London, London, United Kingdom

**Keywords:** obesity, metabolic syndrome, QTc interval, ventricular arrhythmia, polygenic risk score

## Abstract

**Background:**

Small-scale studies have linked obesity (Ob) and metabolic ill-health with proarrhythmic repolarisation abnormalities. Whether these are observed at a population scale, modulated by individuals’ genetics, and confer higher risks of ventricular arrhythmias (VA) are not known.

**Methods and Results:**

Firstly, using the UK Biobank, the association between adiposity and QTc interval was assessed in participants with a resting 12-lead ECG (*n* = 23,683), and a polygenic risk score (PRS) was developed to investigate any modulatory effect of genetics. Participants were also categorised into four phenotypes according to the presence (+) or absence (–) of Ob, and if they were metabolically unhealthy (MU+) or not (MU-). QTc was positively associated with body mass index (BMI), body fat (BF), waist:hip ratio (WHR), and hip and waist girths. Individuals’ genetics had no significant modulatory effect on QTc-prolonging effects of increasing adiposity. QTc interval was comparably longer in those with metabolic perturbation without obesity (Ob-MU+) and obesity alone (Ob+MU-) compared with individuals with neither (Ob-MU-), and their co-existence (Ob+MU+) had an additive effect on QTc interval. Secondly, for 502,536 participants in the UK Biobank, odds ratios (*OR*s) for VA were computed for the four clinical phenotypes above using their past medical records. Referenced to Ob-MU-, ORs for VA in Ob-MU+ men and women were 5.96 (95% CI: 4.70–7.55) and 5.10 (95% CI: 3.34–7.80), respectively. ORs for Ob+MU+ were 6.99 (95% CI: 5.72–8.54) and 3.56 (95% CI: 2.66–4.77) in men and women, respectively.

**Conclusion:**

Adiposity and metabolic perturbation increase QTc to a similar degree, and their co-existence exerts an additive effect. These effects are not modulated by individuals’ genetics. Metabolic ill-health is associated with a higher OR for VA than obesity.

## Introduction

Obesity is a growing international health burden (1). Although there is compelling evidence for the direct adverse effect of increasing adiposity on atrial arrhythmogenesis (2), the evidence for any associations between obesity and ventricular arrhythmogenesis is less robust. Small-scale studies have shown obesity to be associated with QT (QTc) interval prolongation (3), but whether this is also true at a population-level and, importantly, if this translates into a higher risk for ventricular arrhythmias (VA), has not been fully established.

Obesity often co-exists with modifiable cardiovascular risk factors, such as insulin resistance, hypertension, and dyslipidaemia, the combination of which is referred to as metabolic syndrome (4). This has led to the recognition of distinct obesity phenotypes, defined by the presence or absence of concomitant adverse metabolic profiles (5). Although metabolic perturbation is also associated with ventricular repolarisation abnormalities (6), it is unknown if the effects of obesity and metabolic ill-health on proarrhythmic ventricular remodelling are additive.

Myocardial repolarisation is partly genetically determined, with at least 85 single nucleotide polymorphisms (SNPs) shown to be associated with QTc interval (7). An individual’s propensity for QTc prolongation is therefore influenced by the unique expression of SNPs contributing toward myocardial repolarisation (8). Whether genetics modulate any associations between increasing adiposity and/or metabolic ill-health and QTc, respectively, also remains unknown.

The UK Biobank is an ongoing longitudinal study that has recorded electrocardiographic (ECG), clinical and, uniquely, genetic data amongst its participants since 2006 (9). It is ideally placed to address the limitations in our understanding of how increasing adiposity and metabolic perturbations adversely alter ventricular repolarisation. Leveraging this resource, first we sought to establish the associations between obesity and metabolic dysfunction, respectively, with QTc interval, at a population-level. Second, we determined whether these associations are modulated by individuals’ genetic makeup. Finally, we compared the odds for VA amongst individuals with obesity and/or metabolic ill-health.

## Materials and Methods

Over 500,000 participants aged 40–69 years were recruited to the UK Biobank at 22 assessment centres between 2006 and 2010 (9). It was conducted in accordance with relevant guidelines and regulations, with approval from the North West Multicentre Research Ethics Committee. Participants provided informed consent for their data to be used for health-related research. Data were accessed under application numbers 48666 and 47602. This study conforms to the Declaration of Helsinki.

### Clinical Definitions and Diagnoses

The indices of adiposity considered in the analyses were body mass index (BMI, kg/m^2^), body fat percentage (BF,%), waist:hip ratio (WHR, unit), hip girth (HG, cm), and waist girth (WG, cm). Obesity was defined as BMI ≥ 30 kg/m^2^, or WG > 94 cm in men and > 80 cm in women (4, 10). Recorded diagnoses of any two of dyslipidaemia, hypertension, or type 1 or 2 diabetes mellitus based on International Classification of Diseases (ICD)-10 classifications (Supplementary Table 1) in a participant’s healthcare records categorised them as “metabolically unhealthy”. We reviewed biochemistry and blood pressure results of individuals in whom there was no recorded medical history of dyslipidaemia or hypertension. These individuals were assigned a diagnosis of dyslipidaemia or hypertension in our analyses if their results met the criteria for metabolic syndrome outlined in the interim statement from the International Diabetes Federation (4). In these cases, dyslipidaemia was defined as either raised triglycerides [>150 mg/dL (1.7 mmol/L)] or reduced high-density lipoprotein (HDL) cholesterol [<40 mg/dL (1.03 mmol/L) for men and <50 mg/dL (129 mmol/L) in women]. Hypertension was defined as having a systolic blood pressure ≥130 mmHg or a diastolic blood pressure ≥85 mmHg. Individuals were thereby identified as obese (Ob+), non-obese (Ob-), metabolically unhealthy (MU+), or metabolically healthy (MU-) and separated into four phenotypes: Ob-MU-, Ob-MU+, Ob+MU-, and Ob+MU+. The incidence of VA was determined based on the presence of at least one ICD-10 code for the condition listed as a primary or secondary diagnosis, or an intervention at any time in an individual’s medical record. VA was defined as ventricular tachycardia (VT), ventricular fibrillation (VF), re-entry ventricular arrhythmia, and catheter ablation of the ventricular wall (Supplementary Table 1).

### Effect of Increasing Adiposity and Obesity Phenotypes on QTc Interval

This analysis was confined to participants that had a recorded QTc by Bazzett’s formula from a single resting 12-lead ECG (GE Cardiosoft), recorded at a UK Biobank assessment centre, and anthropometry in the same window of recruitment to the UK Biobank (2015–2017, *n* = 23,683) by a trained technician. The exception were BF data that were only recorded in the first round of recruitment to the UK Biobank (starting 2006). Adjusted multiple linear regression analyses were performed in two models to determine the effect of increasing adiposity on QTc. Model 1 was adjusted for age, sex, ethnicity, Townsend deprivation index (TDI, as shown in Supplementary Methods) and model 2 further adjusted for hypertension, dyslipidaemia, coronary artery disease, stroke, diabetes, smoking, and alcohol consumption as dichotomous variables listed in participants past medical records or self-reported questionnaires (Supplementary Table 1). The indices of adiposity were treated as continuous variables to determine their effects on QTc for every unit and standard deviation (SD) increment in adiposity. A similar analysis adjusted for age, sex, ethnicity, and TDI was used to assess the effect of clinical phenotypes on QTc.

### Polygenic Risk Score for QTc Interval Prolongation

Polygenic risk scores (PRS) provide a quantitative metric of an individual’s inherited risk based on the cumulative impact of many common variants. The derivation of PRS for QT interval is included in Supplementary Table 2. A high PRS represents a greater genetic propensity for a longer than normal QTc interval than a low PRS. We therefore considered PRS a surrogate, and inverse, measure of an individual’s inherent repolarisation reserve. To study the modulatory effect of genetics on the association between increasing adiposity and QTc amongst individuals that had been genotyped, PRS was added to model 2 to generate model 3 in the regression analysis. Additionally, we stratified individuals into high (> 75%), intermediate (25–75%), and low (< 25%) PRS groups, representing low, intermediate, and high repolarisation reserve, respectively, to identify any differential effects of adiposity on QTc between these groups. The modulatory effect of PRS for QT interval on the associations between QTc and obesity and metabolic perturbation, respectively, was also investigated.

### Effect of Obesity and Metabolic III-Health on Ventricular Arrhythmias

Ventricular arrhythmias occurred infrequently amongst the UK Biobank’s subpopulation in which 12-lead ECG data were available (*n* = 23,683). We therefore investigated the association between obesity phenotypes and VA across the whole UK Biobank (*n* = 502,536). For the purposes of this analysis, an individual’s mean BMI or WG was used to define obesity (BMI ≥ 30 kg/m^2^ or WG > 94 cm in men and > 80 cm in women) (4) in instances where this data had been recorded more than once.

### Statistical Analyses

Continuous variables are expressed as mean ± SD. All confidence intervals (CI) are provided as a 95% range. The associations between indices of adiposity and QTc were modelled using multivariable linear regression models. Logistic regression adjusted for age, sex, ethnicity, and TDI was used to assess associations between obesity phenotypes and QTc and VA, respectively. Analyses were performed using with Python version 3.6 (Python Software Foundation). The value of *p*< 0.05 was considered significant.

## Results

In total, 23,683 participants (11,563 men, mean age 61.0 ± 7.5years, and 97.1% Caucasian) had ECG, BMI, or at least one other anthropometric measurement, along with sociodemographic and ICD-10 information. Table 1 provides characteristics for the UK Biobank participants in whom the effects of adiposity and clinical phenotypes on QTc were investigated.

**TABLE 1:** The characteristics of the UK Biobank participants in whom the effects of adiposity and clinical phenotype on QTc interval was assessed.

	Total	Ob–MU–	Ob+MU-	Ob–MU+	Ob+MU+
**N**	23,683	10,500	8,818	1,571	2,794
**Sex**					
Male	11,563 (48.8%)	5,219 (49.7%)	3,695 (41.9%)	1,099 (70.0%)	1,550 (55.5%)
Female	12,120 (51.2%)	5,281 (50.3%)	5,123 (58.1%)	472 (30.0%)	1,244 (44.5%
**Age (years)**	61.0 + 7.5	59.9 + 7.5	60.4 + 7.4	65.0 + 6.5	64.4 + 6.6
**Ethnicity**					
Non-Caucasian	647 (2.7%)	313 (3.0%)	190 (2.2%)	63 (4.0%)	81 (2.9%)
Caucasian	22,996 (97.1%)	10,169 (96.8%)	8,613 (97.7%)	1,504 (95.7%)	2,710 (97.0%)
Non-specified	40 (0.2%)	18 (0.2%)	15 (0.2%)	4 (0.3%)	3 (0.1%)
**Townsend deprivation index (units)**	–2.0 + 2.7	–2.2 + 2.6	–1.9 + 2.7	–2.1 + 2.7	–1.8 + 2.8
**Smoking status**					
Current	914 (3.9%)	404 (3.8%)	349 (4.0%)	49 (3.1%)	112 (4.0%)
Previous	7,947 (33.6%)	3,056 (29.1%)	3,058 (34.7%)	608 (38.7%)	1,225 (43.8%)
Never/	14,616 (61.7%)	6,970 (66.4%)	5,326 (60.4%)	900 (57.3%)	1,420 (50.8%)
Prefer not to answer	69 (0.3%)	20 (0.2%)	29 (0.3%)	7 (0.4%)	13 (0.5%)
Non-specified	137 (0.6%)	50 (0.5%)	56 (0.6%)	7 (0.4%)	24 (0.9%)
**Alcohol consumption**					
Current	21,999	9,843	8,202	1,434	2,520
Previous	762	293	263	67	139
Never	778	312	294	63	109
Prefer not to answer	7	2	3	0	2
Non-specified	137	50	56	7	24
**QTc interval (ms)**	420.4 + 26.6	416.5 + 25.0	422.9 + 27.5	420.6 + 26.7	427.2 + 27.0
**Heart rate (bpm)**	60 + 9	58 + 8	62 + 8	60 + 9	63 + 10
**Anthropometry**					
BMI (kg/m^2^)	26.7 + 4.5	23.8 + 2.4	29.2 + 4.1	24.6 + 2.4	30.6 + 4.5
WG (cm)	88.4 + 12.6	79.8 + 8.5	95.7 + 10.1	83.7 + 7.8	100.1 + 11.1
HG (cm)	101.4 + 8.8	96.1 + 5.4	106.6 + 8.1	96.5 + 5.0	107.8 + 8.9
WHR (unit)	0.87 + 0.09	0.83 + 0.08	0.90 + 0.08	0.87 + 0.07	0.93 + 0.08
BF (%)	30.1 + 8.2	26.3 + 7.0	34.0 + 7.5	26.1 + 6.4	34.3 + 7.9

*Values presented as mean [±standard deviation (SD)]. Percentages of the cohort in each category presented in parentheses. Ob, obesity; MU, metabolically unhealthy; +, presence; – absence; BMI, body mass index (kg/m^2^); BF, body fat (%); WHR, waist:hip ratio (unit); HG, hip girth (cm); WG, waist girth (cm).*

### Increasing Adiposity Is Associated With QTc Interval

**FIGURE 1:**
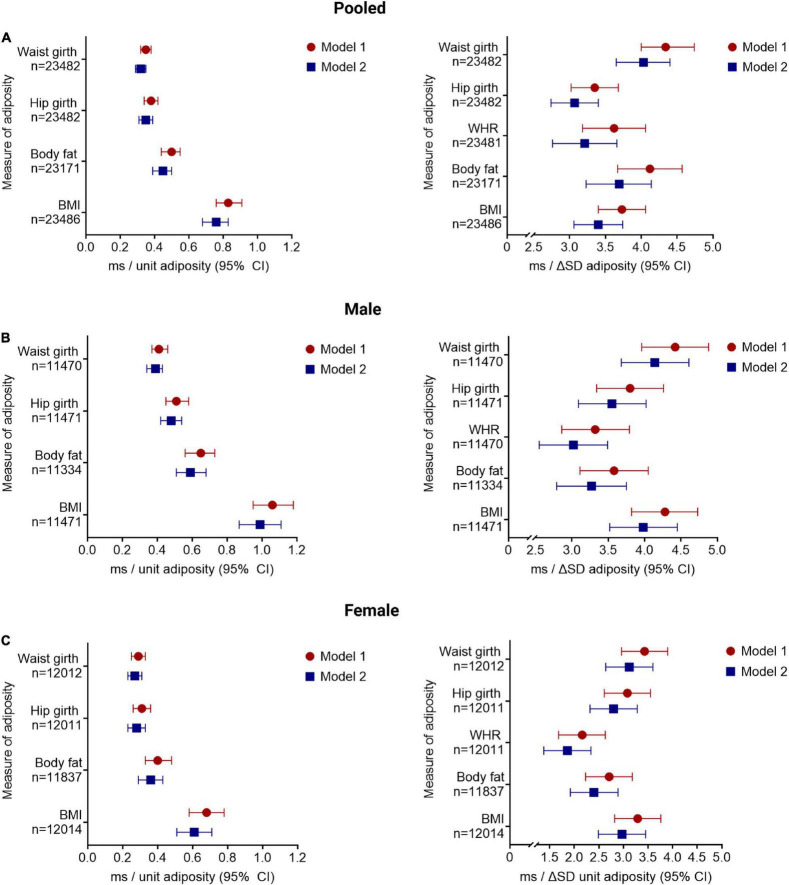
Increasing adiposity is associated with increased QTc interval, after adjusting for sociodemographic, co-morbidity, and lifestyle factors. The change in QTc per unit and per standard deviation (SD) increment of in adiposity [quantified by BMI, body mass index (kg/m^2^); BF, body fat (%); WHR, waist:hip ratio (unit); HG, hip girth (cm); and WG, waist girth (cm)] are shown for the pooled cohort **(A)** and stratified by sex **(B,C)**. Model 1 adjusted for sociodemographic factors; model 2 adjusted for lifestyle and comorbidities. All results presented reached significance *p* < 0.001. Results for QTc change per unit increment in WHR is provided in the main text.

There was a positive association between the QTc interval and adiposity, quantified by the listed anthropometric measurements (Figure 1). For the pooled analysis (*n* = 23,482), correcting for sociodemographic factors (model 1), the QTc interval was increased by 0.83 ms/kg/m^2^ (BMI, CI: 0.76–0.91 ms/kg/m^2^), 0.50 ms/% BF (CI: 0.44–0.55 ms/%), 42.10 ms/unit WHR (CI: 36.95–47.26 ms/unit), 0.38 ms/cm HG (CI: 0.34–0.42 ms/cm), and 0.35 ms/cm WG (CI: 0.32–0.38 ms/cm; all *p* < 0.001).

The QTc interval increases, albeit to a lesser degree, with increasing adiposity when additionally adjusted for co-morbidity and lifestyle factors (model 2): 0.76 ms/kg/m^2^ BMI (CI: 0.68–0.83 ms/kg/m^2^), 0.45 ms/% BF (CI: 0.39–0.50 ms/%), 37.34 ms/unit WHR (CI: 32.10–42.58 ms/unit), 0.35 ms/cm HG (CI: 0.31–0.39 ms/cm), and 0.32 ms/cm WG (CI: 0.29–0.35 ms/cm; all *p* < 0.001). Similar observations were noted in sex-stratified analyses (Figure 1).

### Genetically Determined QT Interval Does Not Modulate the Associations Between Adiposity and QTc

**FIGURE 2:**
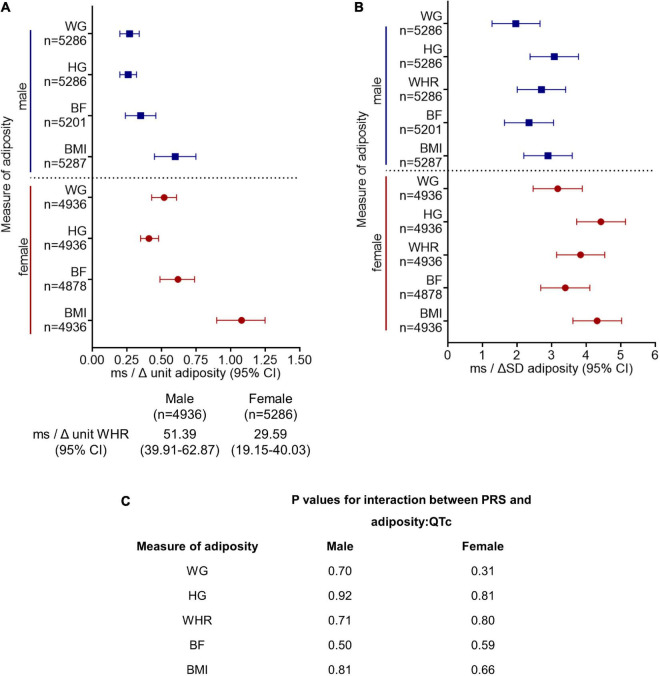
Polygenic risk score (PRS) for genetically determined QT interval does not modulate the association between increasing adiposity and QTc interval. The change in QTc per unit **(A)** and per standard deviation (SD) **(B)** increment in adiposity [quantified by BMI, body mass index (kg/m^2^); BF, body fat (%); WHR, waist:hip ratio (unit); HG, hip girth (cm); WG, waist girth (cm)] are shown stratified by sex. Model 3 is adjusted for PRS in addition to sociodemographic and lifestyle factors and comorbidities as in model 2 (Figure 1). All results presented for model 3 in **(A,B)** reached significance *p* < 0.001. **(C)** There was no significant interaction between PRS and the adiposity:QTc relationships (P-interaction, PRS with adiposity:QTc) in either sex, suggesting genetics do not modulate the relationship between adiposity and QTc.

**FIGURE 3:**
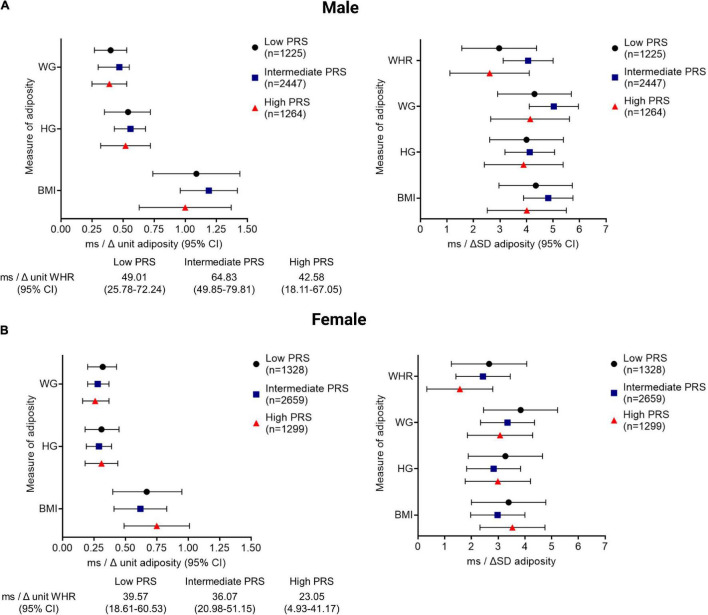
The QTc interval prolongs comparably with increasing adiposity in men and women stratified by PRS. The change in the QTc per unit and per SD increment of adiposity [quantified by BMI, body mass index (kg/m^2^); HG, hip girth (cm); WG, waist girth (cm); WHR, waist; hip ratio (unit)] is shown stratified according to low (<25%), intermediate (25–75%), and high (>75%) PRS, representing high, intermediate, and low repolarisation reserve, respectively for men **(A)** and women **(B)**. All associations between QTc and adiposity measures reached statistical significance with *p* < 0.001.

Amongst those individuals that had ECG, anthropometry, and genotype data available (*n* = 10,223), there was a positive correlation between QTc and PRS (*r* = 0.17, *p* < 0.001), such that QTc increased by 4.50 ms (CI: 4.00–5.02 ms) per unit increment in the PRS for genetically determined QT interval in the pooled cohort. QTc increased by 4.33 ms (CI: 3.64–5.04 ms) and 4.74 ms (CI: 4.03–5.46 ms) per unit increment in PRS in women and men, respectively (both *p* < 0.001). QTc was observed to be greater with every unit and SD increment in adiposity in men and women, after adjusting for the PRS, in addition to socio-demographic, co-morbidity, and lifestyle factors (model 3, Figure 2). However, the interactions between PRS and the adiposity-QTc relationships were not statistically significant (Figure 2). Furthermore, there was no consistent evidence to show that the associations between indices of adiposity and QTc were modulated by PRS when stratified by PRS in men or women (Figure 3).

### Metabolic Perturbation and Obesity Exert Additive Effects on QTc Interval

**FIGURE 4:**
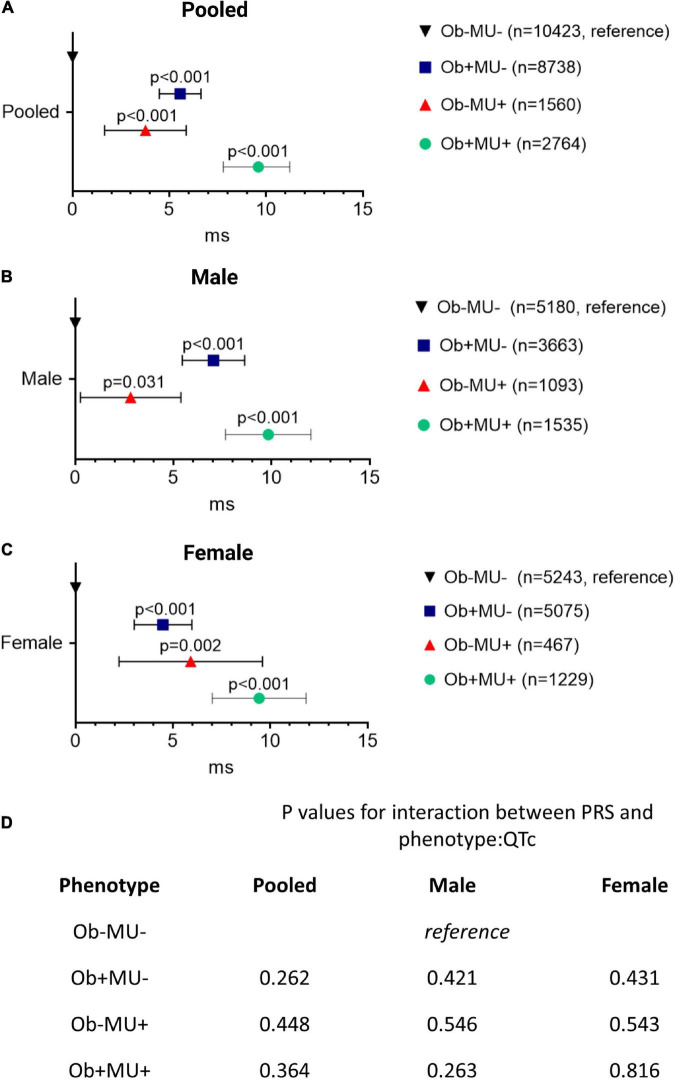
Obesity and metabolic perturbation are independently associated with longer QTc interval and their effects are additive when they co-exist. Differences in the QTc interval (ΔQTc) referenced to the non-obese metabolically healthy (Ob-MU-) group for each clinical phenotype, in pooled **(A)** and sex-stratified **(B,C)** analysis. The *p*-values for the interactions between PRS and phenotype:QTc are shown in the in-set table **(D)** Ob, obese; MU, metabolically unhealthy; +, presence; –, absence; CI, confidence interval; PRS, polygenic risk score.

Referenced to Ob-MU-, and after adjusting for sociodemographic factors, both obesity and metabolic perturbation were independently associated with longer QTc intervals in the pooled cohort (Ob+MU- + 5.56 ms, CI: 4.48–6.64 ms; Ob-MU+ + 3.77 ms, CI: 1.66–5.87 ms, both *p <* 0.001; Figure 4). The effects of obesity and metabolic perturbation on QT interval were additive, with concurrent obesity and metabolic perturbation (Ob+MU+) being associated with the longest QTc (+ 9.60 ms, CI: 7.79–11.22 ms, *p* < 0.001). Similar observations were noted in sex-stratified analyses for men (*n* = 11,471) and women (*n* = 12,014) (Figure 4). These associations were not modulated by PRS for genetically determined QT interval (all *p* > 0.05, Figure 4).

### Metabolic Perturbation Exerts a Greater Effect Than Obesity on Ventricular Arrhythmias Risk

Table 2 shows the characteristics for the UK Biobank cohort (*n* = 502,536, male 223,477, mean age 62.5 + 8.1 years, and 94.1% Caucasian) stratified by clinical phenotypes in which VA risk was assessed. Odds ratios (*OR*s) for VA were calculated after addressing the imbalance between numbers of individuals with and without a history of VA; individuals without VA were down-sampled and randomly selected to achieve a ratio of 4:1 (no VA:VA).

**TABLE 2:** The characteristics of the UK Biobank participants stratified by clinical phenotypes in whom risk of ventricular arrhythmias was assessed.

	Total	Ob-MU-	Ob+MU-	Ob-MU+	Ob+MU+
N	502,536	173,308	193,669	35,355	100,204
**Sex**					
Male	223,477	76,968	76,033	21,041	49,435
Female	264,811	95,634	108,374	13,951	46,852
**Age (years)**	62.5 + 8.1	60.3 + 8.2	61.7 + 8.0	66.3 + 6.9	66.2 + 6.8
**Ethnicity**					
Caucasian	472,793	163,880	182,939	32,666	93,308
Non-Caucasian	22,996	10,169	8,613	1,504	2,710
Non-specified	2,717	920	886	314	597
**Townsend deprivation index (units)**	–1.3 + 3.1	–1.5 + 3.0	–1.3 + 3.1	–1.3 + 3.2	–0.9 + 3.2
**No ventricular arrhythmia**	483,014	171,382	183,533	33,899	94,200
**Ventricular arrhythmia**	3,279	456	543	592	1,688

*Values presented as mean (±SD). Ob, obesity; MU, metabolically unhealthy; +, presence; – absence.*

In both men and women, obesity alone (Ob+MU-) was not associated with a higher incidence of VA compared with non-obese healthy (Ob-MU-) individuals (Table 3). On the contrary, metabolic ill-health alone (Ob-MU+) was associated with more VA compared with Ob-MU- (men *OR* 5.96, CI: 4.70–7.55), women *OR* 5.10, CI: 3.34–7.80; both *p* < 0.001). Compared with Ob-MU-, metabolically unhealthy obese (Ob+MU+) men were more likely to have documented VA (*OR* 6.99, CI: 5.72–8.54, *p <* 0.001); in Ob+MU+ women the *OR* for VA was 3.56 (CI: 2.66–4.77; *p* < 0.001). The history of VA was more frequent in Ob+MU+ men than in metabolically unhealthy non-obese (Ob-MU+) men (*OR* 1.25, CI: 1.05–1.49, *p* = 0.036), but not in women (*OR* 1.16, CI: 0.80–1.68, *p* = 0.315). In males, obesity was associated with an increased VA incidence in the context of metabolic ill-health.

**TABLE 3:** Metabolic perturbation confers a greater risk of VA than obesity in men and women.

Reference	Ob+MU-	Ob-MU+	Ob+MU+
**Male**			
Ob-MU-	1.10 (0.87–1.39) *p* = 0.203	5.96 (4.70–7.55) *p* < 0.001	6.99 (5.72–8.54) *p* < 0.001
Ob+MU-	–	–	6.01 (4.98–7.26) *p* < 0.001
Ob-MU+	–	–	1.25 (1.05–1.49) *p* = 0.036
**Female**			
Ob-MU-	0.87 (0.64–1.18) *p* = 0.326	5.10 (3.34–7.80) *p* < 0.001	3.56 (2.66–4.77) *p* < 0.001
Ob+MU-	–	–	5.61 (4.18–7.52) *p* < 0.001
Ob-MU+	–	–	1.16 (0.80–1.68) *p* = 0.315

*The odds ratios (ORs) for VA referenced to clinical phenotypes, stratified by sex and adjusted for age, ethnicity and Townsend deprivation index (TDI), are shown. Metabolic ill-health alone confers greater risks of VA than obesity alone. For male, obesity adds to the risk of VA in the context of metabolic ill-health. Ob, obese; MU, metabolically unhealthy; +, presence; –, absence; 95% confidence intervals in parentheses.*

## Discussion

Using UK Biobank data, we examined the associations between adiposity and metabolic ill-health, respectively, with QTc, and investigated the burden of VA amongst obesity phenotypes. The main findings are: (i) QTc is longer in individuals with higher BMI and greater central adiposity after adjusting for sociodemographic, lifestyle factors, and co-morbidity; (ii) genetically-determined QT interval, assessed using PRSs, does not modulate the QTc-prolonging effects of adiposity; (iii) metabolic perturbation and obesity are independently associated with longer QTc, and this effect is additive when they co-exist; and (iv) metabolic perturbation alone is associated with a greater incidence of VA than obesity alone, though obesity confers additional VA risks in the context of metabolic ill-health in men.

### Increasing Adiposity Is Associated With QTc Interval

We demonstrate an association between increasing adiposity and a QTc interval that is independent of comorbidity and lifestyle factors. Although BMI is the most common metric to categorise obesity, it is indiscriminate between fat and muscle and their respective distributions. Therefore, we analysed other anthropometric indices that are better indicators of central (visceral) adiposity, which have been associated with metabolic syndrome (10) and increased arrhythmic risk (11). In addition, we showed a consistent association between adiposity and QTc prolongation for these additional indices.

There are several mechanisms that may explain the association between increasing adiposity and QTc interval. Adipose tissue is metabolically active and there are compelling data suggesting the visceral depot secretes pro-inflammatory and arrhythmogenic adipocytokines, which modulate ionic channels and prolong ventricular action potential duration (APD). For example, interleukin (IL)-6 increases L-type calcium (I_CaL_) (12) and suppresses rapid delayed rectifier K^+^ (I_Kr_) currents (13). IL-1β reduces the transient outward K^+^ current (I_to_) (14) and increases I_CaL_, also causing APD prolongation (15). Tumour necrosis factor-α (TNF-α) suppresses delayed rectifier K^+^ currents (16) and reduces K_v4.2_ and K_v4.3_ protein and K_v_ channel-interacting protein-2 mRNA expression (17). More specifically, the pericardial fat secretome has been associated with reduced I_Kr_ and increased I_CaL_ currents in metabolic syndrome, resulting in APD prolongation (18). Serum levels of the adipokine fatty-acid binding protein has also been suggested as a biochemical proxy for prolonged QTc interval and reduced ejection fraction in patients with stable angina (19). Similarly, plasma adiponectin may modulate ventricular repolarisation and thereby QTc interval (20). Given that APD prolongation manifests as QTc prolongation on 12-lead ECG, our results are compatible with adipocytokine-mediated modulation of ionic channels.

Aside from the direct proarrhythmic effects of adipose secretome, chronic obesity confers arrhythmic risks *via* systemic metabolic complications, such as non-alcoholic fatty liver disease (NAFLD) (21, 22). NAFLD has been linked to higher rates of adverse cardiovascular outcomes (23) that include greater risks of atrial fibrillation (24–26), QTc prolongation (27, 28), and VA (29, 30). Insulin resistance and dysregulated lipid metabolism resulting in release of pro-inflammatory, -fibrotic and -oxidising hepatic mediators may underpin these observations (22, 31, 32). Furthermore, NAFLD favours epicardial fat accumulation resulting in cardiac lipotoxicity, fatty infiltration, fibrosis, and yet more secretion of inflammatory mediators. That creates a vicious cycle of increasing metabolic perturbation to cause structural, electrical, and autonomic remodelling (33, 34).

### Underlying Genetics Do Not Modulate QTc Prolongation Associated With Adiposity

Genome-wide association study (GWAS) studies suggest that up to 10% of QTc interval variation in a given population can be explained by genetic differences associated with myocardial ionic homeostasis (7). Despite this, the influence of genetic variation on QTc prolongation in the context of obesity and metabolic dysfunction has not been investigated. We generated a PRS based on 66 SNPs available in the UK Biobank that are known to influence QTc (7, 35) as a quantifiable metric of an individual’s genetic propensity for longer than normal QTc, and thereby a surrogate for repolarisation reserve. We did not find any consistent evidence to show that low PRS, as a proxy for high repolarisation reserve, attenuates or protects against any QTc-prolonging effects of increasing adiposity. Furthermore, the interaction of PRS with the relationships between adiposity and QTc was not statistically significant. Our results suggest that excess adiposity is a sufficiently potent extrinsic stressor that overwhelms any protective effect of genetics on QTc prolongation and that modifiable rather than non-modifiable risk factors are more important determinants of QTc interval.

### Metabolic Perturbation Is Associated With QT Prolongation

Amongst the four clinical phenotypes, QTc was the longest in those who are obese and metabolically unhealthy (Ob+MU+) and the shortest in those who have neither (Ob-MU-). Metabolically healthy obese (Ob+MU-) and metabolically unhealthy lean (Ob-MU+) individuals have comparable QTc intervals that were longer than Ob-MU- individuals but shorter than observed in Ob+MU+. These observations held true in sex-stratified analyses. PRS did not modulate the relationships between phenotype and QTc. Our results are consistent with previous small-scale studies and suggest that obesity and metabolic perturbation, whether alone or in combination, are not electrophysiologically benign. For example, metabolic syndrome has been associated with prolonged ventricular repolarisation as a consequence of reduced voltage-gated K^+^ channel current, and greater intracellular Na^+^ and Ca^2+^ concentrations from increased activity of Na^+^/Ca^2+^ and Na^+^/H^+^ exchanger proteins (36).

### Metabolic III-Health Confers a Greater Risk of Ventricular Arrhythmias Than Obesity

We investigated the association between obesity phenotypes and VA in the UK Biobank population stratified by their obesity and metabolic status. Given that obesity is associated with a higher burden of ventricular ectopy (37–39), and a critically timed extrasystole can trigger re-entrant arrhythmias, our definition of VA also included catheter ablation of the ventricular wall in addition to VT/VF. VAs occurred most frequently in the Ob+MU+ phenotype and least frequently in Ob-MU-, suggesting that our four obese phenotypes exist on a spectrum of increasing arrhythmic risk. Referenced to Ob-MU-, the *OR*s of VA in metabolically unhealthy individuals (Ob-MU+) were over fivefold greater in men and women. In both sexes, metabolic dysfunction conferred incremental VA risks in the absence and presence of obesity. Obesity in the context of metabolic dysfunction, i.e., Ob-MU+ vs. Ob+MU+, was associated with an incremental VA risk in men but not women. Although both obesity and metabolic perturbation can increase the risk of VA, the latter appears to have a disproportional effect. These results were observed in the whole UK Biobank cohort that had comparable sociodemographic baseline characteristics to the subset in which the effect of adiposity on QTc interval was assessed. Factors aside from abnormal repolarisation, such as aberrant depolarisation, fibrosis, and increased incidence of structural heart disease and coronary artery disease, may also contribute to arrhythmic risk in this group. For example, diabetic cardiomyopathy is the consequence of chronic activation of renin-angiotensin-aldosterone and sympathetic nervous systems, oxidative stress, and endothelial dysfunction, which promote cardiac tissue interstitial fibrosis and thereby conduction and repolarisation heterogeneity that increases arrhythmia propensity (40). The greater risk of VA in metabolically unhealthy individuals compared with their healthy obese counterparts may also be attributed to a greater and more unstable plaque burden even in non-culprit coronary lesions, resulting in more adverse outcomes (41).

### Limitations

This was a cross-sectional study in which middle-aged Caucasian individuals were over-represented, and therefore these results may not extrapolate to other ethnic groups or ages. Although we used data for over 500,000 individuals where possible in our analyses, obesity phenotypes were unequally represented and ECG data were available for approximately 5% of the UK Biobank cohort. It is possible that our findings may change with a more even distribution amongst these groups and a larger sample size. Similarly, there were insufficient VA episodes amongst the subset of participants with recorded ECGs to ascertain any associations between QTc and VA. Our analyses could not account for any specific effects of medications on QTc owing to the methods by which drug history was recorded in the UK Biobank. Similarly, our results may only be interpreted in the absence of the detailed electrolyte profiles for the subjects included in our analysis. Participants with asymptomatic VA or with VA-related symptoms that did not result in clinical evaluation would not have been included in our analysis. Finally, our assessment of the association between obesity and metabolic syndrome and VA may be influenced by the accuracy of ICD coding that was used to determine medical diagnoses from healthcare records.

## Conclusion

Amongst individuals with recorded ECG and anthropometry data in the UK Biobank, adiposity and metabolic perturbation are both associated with the increased QTc interval after adjusting for confounding variables, and their co-existence has an additive effect. The genetically determined QT interval, assessed by PRS, does not significantly modulate the relationship between adiposity and QTc, suggesting modifiable rather than non-modifiable risk factors are more important determinants of the QTc interval. Despite comparable QTc-prolonging effects of adiposity and metabolic ill-health, metabolic ill-health alone is associated with a greater burden of VA than obesity alone amongst 500,000 individuals in the United Kingdom population, with individuals with both obesity and metabolic ill-health exhibiting the highest burden of VA.

## Data Availability Statement

Data used in this study can be accessed from the UK Biobank by application. Available at: www.ukbiobank.ac.uk.

## Ethics Statement

The UK Biobank was approved by the North West Multicentre Research Ethics Committee. Participants provided informed consent for their data to be used for health-related research. This study conforms to the Declaration of Helsinki.

## Author Contributions

KHKP performed data analysis and wrote the manuscript. XL accessed UK Biobank data and performed data analysis. XX generated the polygenic risk score using genetic data from the UK Biobank. MA, PPP, SP, NSP, and JSW provided critical analyses of the methods and reviewed the manuscript. FSN conceived the research project, reviewed the data analyses, and edited the manuscript. All authors contributed to the article and approved the submitted version.

## Conflict of Interest

The authors declare that the research was conducted in the absence of any commercial or financial relationships that could be construed as a potential conflict of interest.

## Publisher’s Note

All claims expressed in this article are solely those of the authors and do not necessarily represent those of their affiliated organizations, or those of the publisher, the editors and the reviewers. Any product that may be evaluated in this article, or claim that may be made by its manufacturer, is not guaranteed or endorsed by the publisher.
