# Supplementary material for: Increasing Adiposity Is Associated With QTc Interval Prolongation and Increased Ventricular Arrhythmic Risk in the Context of Metabolic Dysfunction: Results From the UK Biobank
Source: Front Cardiovasc Med. 2022 Jun 29;9:939156. doi: 10.3389/fcvm.2022.939156 (PMC9277510; doi:10.3389/fcvm.2022.939156)
Supplement: Supplementary file 1 [file Data_Sheet_1.docx]

**Supplemental Materials:** **Increasing adiposity prolongs QTc interval and increases risk of ventricular arrhythmias in context of metabolic dysfunction: results from the UK Biobank**

**Authors:** Kiran Haresh Kumar Patel* BSc MRCP^1^, Xinyang Li* PhD^1^, Xiao Xu PhD^1^, Lin Sun BSc^1^, Maddalena Ardissino^2^, Prakash Punjabi MS MCh FRCP^3^, Sanjay Purkayastha MD FRCS^3^, Nicholas S Peters MD FRCP^1^, James S Ware MA PhD MRCP^1^, Fu Siong Ng BSc PhD MRCP^1^

*joint first authors

^1^ National Heart and Lung Institute, Imperial College London, United Kingdom

^2^ Imperial College Healthcare NHS Trust, London, United Kingdom

^3^ Imperial College London, United Kingdom

**Corresponding author:**

Dr Fu Siong Ng

4th Floor, ICTEM Building

Imperial College London

72 Du Cane Road

W12 0NN, United Kingdom

E-mail: f.ng@imperial.ac.uk

Phone: +44 (0)20 7594 3614

Word count: 1268

**Conflict of interest:** none declared.

**Supplemental methods**

**Definitions of co-morbidity and rhythm abnormalities**

Morbidity and rhythm abnormality definitions based on the International Classification of Disease (ICD-10) classification were used in the study analyses. These are listed in **Supplemental Table S1** and encompass the conditions recorded in the primary (main) position in the participants’ hospital inpatient records upon entry to the UK Biobank based on review of past medical records. Participants’ baseline demographic characteristics were collected upon enrolment to the UK Biobank, and includes date of birth, sex and index of deprivation (Townsend deprivation index, TDI). TDI is a census-based index of material deprivation that is widely used in health research to establish whether relationships exist with deprivation. In this study, TDI was used as a surrogate index for lifestyle choices that may associated with adverse health outcomes. It was calculated immediately prior to the participant joining the UK Biobank and was based on preceding national census output areas. Each participant was assigned a score corresponding to the output area in which their postcode was located.

**Supplemental Table S1:**  Definitions of comorbidity based on the main and secondary International Classification of Disease (ICD)-10 summary diagnosis or operative procedures.

| Comorbidity | ICD Code | Definition |
| --- | --- | --- |
| Hypertension | I10, I15  (Diagnosis) | Essential (primary) hypertension; Secondary hypertension. |
| Diabetes | E10, E11, E13, E14  (Diagnosis) | Insulin-dependent diabetes mellitus; Non-insulin-dependent diabetes mellitus; Other specified diabetes mellitus; Unspecified diabetes mellitus. |
| Dyslipidaemia | E78.0, E78.1, E78.2, E78.4, E78.5, E78.6  (Diagnosis) | Pure hypercholesterolaemia; Pure hyperglyceridaemia; Mixed hyperlipidaemia; Other hyperlipidaemia; Hyperlipidaemia, unspecified; Lipoprotein deficiency. |
| Coronary artery disease | I20, I21, I22  I24, I25  (Diagnosis) | Angina pectoris; Acute myocardial infarction; Subsequent myocardial infarction; Other acute ischaemic heart diseases; Chronic ischaemic heart disease. |
| Ventricular Arrhythmias | I47.0, I47.2, I49.0  (Diagnosis) | Re-entry ventricular arrhythmia; Ventricular tachycardia; Ventricular fibrillation and flutter; |
|  | K57.6  (Operative Procedures) | Percutaneous transluminal ablation of ventricular wall; |

**Derivation of polygenic risk score (PRS) for QTc interval**

To calculate the PRS for QT interval, we use summary statistics from prior genome-wide association studies of 70K individuals(1). P value threshold at genome-wide association level (P≤5×10^–8^ or –log_10_P=7.3) was used. 67 SNPs passed the threshold and LD clumping (minimum r^2^ = 0.05, distance 500kb) are selected and 66 of them were also available in the UK biobank genotyping dataset. **Supplemental Table S2** displays a list of the SNPs for use in the PRS were created based on increasing significance level from −log_10_P>7.39 (P≤4.07×10^–8^) to - log_10_P>212.9 (P≤1.32×10^–213^). PRS analysis was performed by PRSice (version 2.2.11.b) software using an additive genetic model. PRS score for each UK biobank individual was calculated by β coefficient weighted sum of all 66 selected SNPs and normalized to mean 0 standard deviations 1, as demonstrated by the equation below:

$$PRSj=\frac{\sum i(Si\times Gij)-Mean(PRS)}{SD(PRS)}$$

Where i is ith selected SNP markers used in PRS, j is jth individual, S is β coefficient, G is genotype (coded as 0, 1, and 2) for an additive genetic model.

This 66-SNP QT-PRS explained a significant degree of variance in 14501 UK biobank Caucasian participants for whom QTc was available, with R^2^ =0.0308 in a baseline model examining the association of QTc with PRS. Adding sex and age as covariates, they explained 0.0877 variance of QTc. This result is very similar to the result of the previous report of 2%-5% and 7-8% respectively in the European population(2).

**Supplemental Table S2:** 66 SNPs identified to modulate QT and available in the UK Biobank were used to generate a polygenic risk score as an indicator of repolarisation reserve. Summary statistics from Arking et al (1) were used to derive polygenic risk score. SNP, single nucleotide variants (with corresponding rs identification); CHR, chromosome number; POS: position of each SNP in hg19 reference genome; A1, allele 1; A2, allele 2; HAPMAP_A1_FREQ: A1 allele frequency in Hapmap project; Beta, beta co-efficients from GWAS identifying allele with QT prolongation(1).

| **SNP** | **CHR** | **POS** | **A1** | **A2** | **HAPMAP_A1_FREQ** | **Beta** | **P** |
| --- | --- | --- | --- | --- | --- | --- | --- |
| rs12143842 | 1 | 162033890 | T | C | 0.1892 | 3.5009 | 1.32E-213 |
| rs3934467 | 1 | 162182677 | T | C | 0.1525 | 2.7435 | 2.76E-129 |
| rs880296 | 1 | 162128446 | G | C | 0.2009 | 2.2456 | 1.27E-81 |
| rs11153730 | 6 | 118667522 | T | C | 0.4914 | -1.6491 | 2.23E-67 |
| rs16857031 | 1 | 1162112910 | G | C | 0.1525 | 2.3653 | 6.45E-61 |
| rs37055 | 16 | 58560775 | T | C | 0.2895 | -1.7297 | 2.20E-57 |
| rs7122937 | 11 | 2486550 | T | C | 0.2105 | 1.9273 | 1.24E-54 |
| rs2072413 | 7 | 150647969 | T | C | 0.2931 | -1.6757 | 1.32E-49 |
| rs6676438 | 1 | 161983089 | T | C | 0.2902 | 1.5168 | 2.59E-45 |
| rs846111 | 1 | 6279370 | C | G | 0.2917 | 1.7317 | 7.39E-40 |
| rs347273 | 1 | 162317513 | G | A | 0.07589 | 1.8041 | 1.87E-38 |
| rs3807375 | 7 | 150667210 | T | C | 0.3795 | 1.2227 | 2.88E-33 |
| rs1805120 | 7 | 150649531 | A | G | 0.2054 | 1.5387 | 3.55E-33 |
| rs10919070 | 1 | 169099037 | C | A | 0.1417 | -1.6781 | 1.11E-31 |
| rs735951 | 16 | 11693536 | A | G | 0.4688 | -1.1529 | 2.29E-28 |
| rs12271931 | 11 | 2478519 | G | A | 0.1116 | -2.7657 | 8.94E-28 |
| rs6793245 | 3 | 38599037 | A | G | 0.2857 | -1.1163 | 4.43E-27 |
| rs1052536 | 17 | 33331575 | C | T | 0.4955 | 0.98 | 6.21E-25 |
| rs12210733 | 6 | 118653075 | A | G | 0.03704 | -2.051 | 1.13E-22 |
| rs12061601 | 1 | 169070450 | C | T | 0.1071 | -1.4096 | 3.10E-21 |
| rs4656345 | 1 | 1619912378 | A | G | 0.1 | -4.7305 | 1.03E-19 |
| rs457162 | 6 | 118535983 | T | A | 0.01887 | -1.8493 | 2.49E-18 |
| rs545833 | 1 | 168689940 | T | C | 0.2679 | 0.8975 | 3.89E-17 |
| rs17460657 | 1 | 162261826 | C | A | 0.03333 | -4.6017 | 2.72E-16 |
| rs3902035 | 6 | 119000232 | C | T | 0.1698 | -0.8519 | 8.00E-16 |
| rs1983546 | 1 | 139446183 | G | A | 0.35 | -0.8069 | 9.74E-16 |
| rs2298632 | 1 | 23710475 | T | C | 0.4955 | 0.6998 | 1.38E-14 |
| rs9892651 | 17 | 64303793 | C | T | 0.4688 | -0.7441 | 2.71E-14 |
| rs12997023 | 2 | 40752982 | C | T | 0.05085 | -1.6892 | 4.67E-14 |
| rs7545047 | 1 | 162191103 | A | G | 0.025 | -1.7784 | 8.89E-14 |
| rs12927050 | 16 | 11673488 | A | C | 0.12 | -0.973 | 1.17E-13 |
| rs1811815 | 11 | 2475150 | A | G | 0.2273 | -0.9 | 1.18E-13 |
| rs11710077 | 3 | 38657899 | T | A | 0.1964 | 0.9224 | 1.42E-13 |
| rs10775360 | 17 | 68325868 | T | C | 0.3051 | -0.7551 | 1.07E-12 |
| rs12444261 | 16 | 11734642 | T | G | 0.2857 | -0.7915 | 2.14E-12 |
| rs3105593 | 15 | 50845018 | T | C | 0.4732 | 0.6643 | 3.06E-12 |
| rs6599234 | 3 | 38715300 | A | T | 0.3 | 0.6986 | 2.22E-11 |
| rs11708996 | 3 | 38633923 | C | G | 0.2 | -0.9238 | 2.27E-11 |
| rs164133 | 1 | 162381288 | C | G | 0.1833 | 0.7195 | 2.99E-11 |
| rs2273905 | 14 | 102974999 | T | C | 0.2946 | 0.6102 | 4.04E-11 |
| rs236523 | 17 | 68212642 | C | A | 0.4955 | 0.6377 | 4.77E-11 |
| rs1659127 | 16 | 14388305 | A | G | 0.3 | 0.7068 | 6.38E-11 |
| rs13355516 | 5 | 137380603 | G | A | 0.175 | -0.8306 | 6.97E-11 |
| rs6800541 | 3 | 38774832 | C | T | 0.4196 | -0.6249 | 8.14E-11 |
| rs12079745 | 1 | 139101060 | A | G | 0.02586 | -1.3408 | 1.22E-10 |
| rs174577 | 11 | 61604814 | A | C | 0.3393 | -0.6523 | 1.30E-10 |
| rs6544311 | 2 | 40353277 | A | C | 0.3776 | 0.6457 | 1.79E-10 |
| rs1296720 | 16 | 3873642 | C | A | 0.2455 | 0.8297 | 3.57E-10 |
| rs4716056 | 6 | 16278390 | G | A | 0.3646 | 0.648 | 3.95E-10 |
| rs2301696 | 11 | 2426984 | G | C | 0.4 | -1.1423 | 4.26E-10 |
| rs2363719 | 4 | 72138216 | A | G | 0.09375 | 0.9677 | 7.84E-10 |
| rs12675772 | 8 | 71213705 | G | C | 0.1 | 0.9447 | 1.76E-09 |
| rs11888462 | 2 | 201160499 | T | G | 0.4153 | 0.5605 | 4.95E-09 |
| rs183993 | 4 | 95130025 | G | A | 0.35 | 0.5662 | 5.19E-09 |
| rs13002675 | 2 | 174739352 | A | C | 0.1937 | 0.7217 | 5.38E-09 |
| rs4784934 | 16 | 58459926 | A | G | 0.2091 | 0.6748 | 5.55E-09 |
| rs2273042 | 1 | 6149122 | A | G | 0.1308 | 0.9375 | 6.80E-09 |
| rs4630352 | 12 | 110747419 | A | G | 0.3227 | 0.5675 | 1.09E-08 |
| rs4657172 | 1 | 162179632 | C | G | 0.1583 | -0.8141 | 1.27E-08 |
| rs9489510 | 6 | 119043898 | G | A | 0.3036 | 0.6085 | 1.36E-08 |
| rs728926 | 13 | 74513122 | T | C | 0.3438 | 0.5746 | 2.06E-08 |
| rs9920 | 7 | 116200092 | C | T | 0.07658 | 0.7899 | 2.60E-08 |
| rs2485376 | 10 | 104050006 | A | G | 0.4286 | -0.5629 | 2.69E-08 |
| rs3789530 | 1 | 6153406 | T | C | 0.1205 | 0.9171 | 3.29E-08 |
| rs295449 | 3 | 47375955 | G | A | 0.433 | -0.549 | 3.83E-08 |
| rs2916558 | 8 | 103847399 | A | C | 0.3833 | 0.5563 | 4.07E-08 |

**Supplemental References**

1. Arking DE, Pulit SL, Crotti L, van der Harst P, Munroe PB, Koopmann TT, Sotoodehnia N, Rossin EJ, Morley M, Wang X, Johnson AD, Lundby A, Gudbjartsson DF, Noseworthy PA, Eijgelsheim M, Bradford Y, Tarasov KV, Dorr M, Muller-Nurasyid M, Lahtinen AM, Nolte IM, Smith AV, Bis JC, Isaacs A, Newhouse SJ, Evans DS, Post WS, Waggott D, Lyytikainen LP, Hicks AA, Eisele L, Ellinghaus D, Hayward C, Navarro P, Ulivi S, Tanaka T, Tester DJ, Chatel S, Gustafsson S, Kumari M, Morris RW, Naluai AT, Padmanabhan S, Kluttig A, Strohmer B, Panayiotou AG, Torres M, Knoflach M, Hubacek JA, Slowikowski K, Raychaudhuri S, Kumar RD, Harris TB, Launer LJ, Shuldiner AR, Alonso A, Bader JS, Ehret G, Huang H, Kao WH, Strait JB, Macfarlane PW, Brown M, Caulfield MJ, Samani NJ, Kronenberg F, Willeit J, Smith JG, Greiser KH, Meyer Zu Schwabedissen H, Werdan K, Carella M, Zelante L, Heckbert SR, Psaty BM, Rotter JI, Kolcic I, Polasek O, Wright AF, Griffin M, Daly MJ, Arnar DO, Holm H, Thorsteinsdottir U, Denny JC, Roden DM, Zuvich RL, Emilsson V, Plump AS, Larson MG, O'Donnell CJ, Yin X, Bobbo M, D'Adamo AP, Iorio A, Sinagra G, Carracedo A, Cummings SR, Nalls MA, Jula A, Kontula KK, Marjamaa A, Oikarinen L, Perola M, Porthan K, Erbel R, Hoffmann P, Jockel KH, Kalsch H, Nothen MM, den Hoed M, Loos RJ, Thelle DS, Gieger C, Meitinger T, Perz S, Peters A, Prucha H, Sinner MF, Waldenberger M, de Boer RA, Franke L, van der Vleuten PA, Beckmann BM, Martens E, Bardai A, Hofman N, Wilde AA, Behr ER, Dalageorgou C, Giudicessi JR, Medeiros-Domingo A, Barc J, Kyndt F, Probst V, Ghidoni A, Insolia R, Hamilton RM, Scherer SW, Brandimarto J, Margulies K, Moravec CE, del Greco MF, Fuchsberger C, O'Connell JR, Lee WK, Watt GC, Campbell H, Wild SH, El Mokhtari NE, Frey N, Asselbergs FW, Mateo Leach I, Navis G, van den Berg MP, van Veldhuisen DJ, Kellis M, Krijthe BP, Franco OH, Hofman A, Kors JA, Uitterlinden AG, Witteman JC, Kedenko L, Lamina C, Oostra BA, Abecasis GR, Lakatta EG, Mulas A, Orru M, Schlessinger D, Uda M, Markus MR, Volker U, Snieder H, Spector TD, Arnlov J, Lind L, Sundstrom J, Syvanen AC, Kivimaki M, Kahonen M, Mononen N, Raitakari OT, Viikari JS, Adamkova V, Kiechl S, Brion M, Nicolaides AN, Paulweber B, Haerting J, Dominiczak AF, Nyberg F, Whincup PH, Hingorani AD, Schott JJ, Bezzina CR, Ingelsson E, Ferrucci L, Gasparini P, Wilson JF, Rudan I, Franke A, Muhleisen TW, Pramstaller PP, Lehtimaki TJ, Paterson AD, Parsa A, Liu Y, van Duijn CM, Siscovick DS, Gudnason V, Jamshidi Y, Salomaa V, Felix SB, Sanna S, Ritchie MD, Stricker BH, Stefansson K, Boyer LA, Cappola TP, Olsen JV, Lage K, Schwartz PJ, Kaab S, Chakravarti A, Ackerman MJ, Pfeufer A, de Bakker PI, Newton-Cheh C. Genetic association study of QT interval highlights role for calcium signaling pathways in myocardial repolarization. Nat Genet 2014;46:826-36.

2. Rosenberg MA, Lubitz SA, Lin H, Kosova G, Castro VM, Huang P, Ellinor PT, Perlis RH, Newton-Cheh C. Validation of Polygenic Scores for QT Interval in Clinical Populations. Circ Cardiovasc Genet 2017;10.
